# Design of Piezoelectric Acoustic Transducers for Underwater Applications

**DOI:** 10.3390/s23041821

**Published:** 2023-02-06

**Authors:** Joo Young Pyun, Young Hun Kim, Kwan Kyu Park

**Affiliations:** Engineering Center, Department of Mechanical Convergence Engineering, Hanyang University, 222 Wangsimni-ro, Seongdong-gu, Seoul 04736, Republic of Korea

**Keywords:** underwater vehicle, piezoelectric ceramic, piezoelectric polymer, flextensional transducer, tonpilz transducer

## Abstract

Interest in underwater transducers has persisted since the mid-1900s. Underwater transducers are designed in various shapes using various materials depending on the purpose of use, such as to achieve high power, improve broadband, and enhance beam steering. Therefore, in this study, an analysis is conducted according to the structural shape of the transducer, exterior material, and active material. By classifying transducers by structure, the transducer design trends and possible design issues can be identified. Researchers have constantly attempted new methods to improve the performance of transducers. In addition, a methodology to overcome this problem is presented. Finally, this review covers old and new research, and will serve as a reference for designers of underwater transducer.

## 1. Introduction

When an underwater vehicle moves, it is necessary to detect and track the location of an external object [1,2]. If the distance to other objects is not recognized, the vehicle stands a risk of collision with them. Accordingly, underwater communication and transducers have been developed to detect external objects [3,4,5]. These transducers must detect motion that is far away; therefore, high-power device specifications are needed [6]. To design a high-power transducer, this study attempted to modify the transducer structure, add materials, change the driving piezoelectric material, and implement a transducer array [7,8,9,10,11,12,13]. Flextensional transducers, which enhance their output by changing their shape, have been studied. Flextensional transducers are classified into convex and concave transducers according to their form, and various classes of transducers, such as classes I–VII transducers, exist. Among them, class V transducers, which have a simple design, have received the most interest from researchers. Among the underwater transducers used in the sonar range, those designed to produce high output in preparation for extreme environments are flextensional transducers. These flextensional transducers are classified according to their external shape. Class I and II generate displacement along the central curvature and produce high output performance. Other parts are similar except for the length of the left and right ends. Class III is a combination of two classes I designed together. After that, class IV transducers appeared. When comparing class I to class VII, most of them are formed with a simple structure, but the class IV transducer is the simplest among them. After the appearance of class IV, which consists only of an elliptical shell and piezoelectric material, class V, which has a wedge-shaped end cap, was studied with more focus on the formation of displacement of the shell. Then, class VI transducers appeared, and finally, class VII transducers evolved from class IV were developed. The contents of the flextensional transducer are located in Section 3.

**Figure 1:**
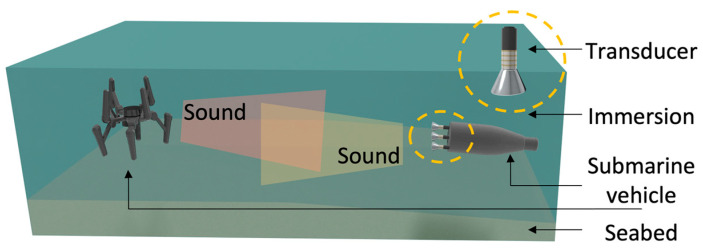
Schematic showing the use of a transducer in water.

Tonpilz transducers are not based solely on a single piezoelectric material but are designed by adding a mass to the front of and behind the piezoelectric material. Another study expanded the transducer bandwidth by changing the physical properties of the piezoelectric material in an additionally designed transducer [14]. Research related to the tonpilz transducer is introduced in Section 4. As research on high power transducers and bandwidths has progressed, considerable attention has been paid to transducer arrays [15]. In Figure 1, the transducer array is attached to the outside of the underwater vehicle on the right-hand side. The attached transducer array consists of tonpilz transducers. Previously, the transducer was designed according to a pitch suitable for the target frequency to form an array; however, recently, more sophisticated arrays have been proposed [14,15]. The transducer is composed of dual layers; thus, each layer has a different resonance. In another example, when designing an array, the resonance frequencies of transducers facing each other were designed. In the transducer array study, a pitch adjustment technique was applied to widen the transducer’s bandwidth [14,15].

Several attempts have been made, and recently, the studied array was attached to an underwater vehicle. Instead of simply arranging an array with identical transducers, an array was formed by combining a transducer for transmitting specialization and a transducer for receiving specialization. Based on these transmission and reception arrays, echo control and coating techniques that are less easily captured by the movement of external objects have also been studied [16,17]. Exposure to external moving objects can be reduced through echo control [17,18]. Consequently, transducers developed in the future are expected to have thin, lightweight, and flexible designs. If the developed transducers are lighter and thinner, the resistance of underwater vehicles can be reduced.

Finally, transducers will continue to be studied; thus, the structural classification of transducers based on piezoelectric materials and types according to transducer material composition is required. Transducers in the sonar range are heavily influenced by their geometry. Therefore, it is classified according to its shape, and the materials that make up the transducers are mentioned, so, we organized and analyzed the transducers belonging to the sonar range. After that, the transmitting performance and receiving performance were summarized. This study serves as a reference for designing transducers in the future, and sheds light on the latest transducer trends. Further, it can help develop a transducer that fits the reader’s purpose.

## 2. Underwater Transducer

Structural changes and various transducer materials have been used to develop transducers for low frequencies. Representative materials used to design transducers include piezoelectric ceramics, composites, and polymers [19,20].

A piezoelectric ceramic is a piezoelectric material, and movement is generated by an applied voltage, which produces sound accordingly. Currently, research has been conducted to widen the bandwidth of the output sound. Piezoelectric ceramics are easy to obtain and exhibit high output pressures but narrow bandwidths [21]. Piezoelectric composite materials have been used to overcome this problem. A piezoelectric composite material is designed by mixing a piezoelectric material with a polymer [22]. The lower the volume fraction of the piezoelectric material, the wider the bandwidth of the output sound. In addition to piezoelectric ceramics and composite materials, piezoelectric polymers have also been used. Piezoelectric polymers have a lower output pressure than that of piezoelectric ceramics and piezoelectric composite materials; however, they are widely used as receiving sensors owing to their wide bandwidth.

**Figure 2:**
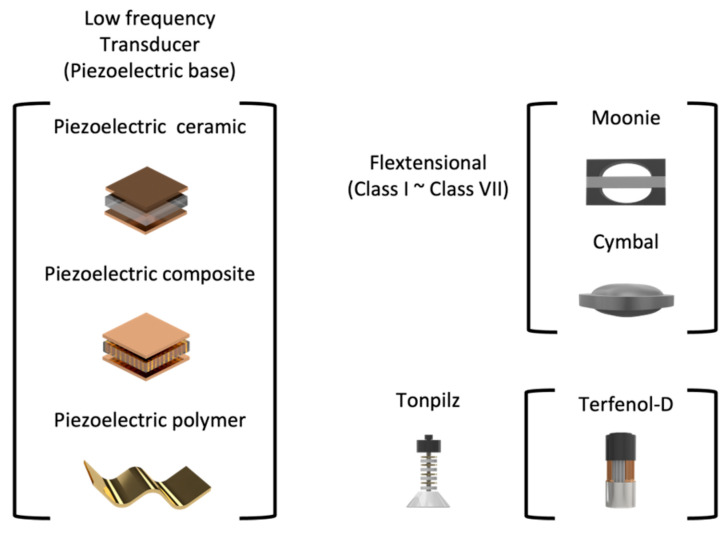
Transducer classification.

Numerous structural designs have been made to fabricate transducers with a high output pressure. As shown in Figure 2, such transducers can be classified as flextensional or tonpilz transducers. Flextensional transducers can be classified into seven types, among which, studies on moonie and cymbal transducers are constantly being conducted. Tonpilz transducers are also being researched for their high output pressure and bandwidth; however, to derive a higher output performance, Terfenol-D has been used as the active material in transducers, or a hybrid method has been introduced.

**Figure 3:**
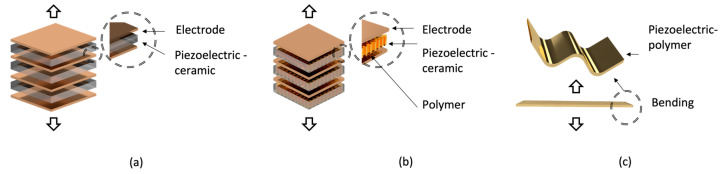
Stacked piezoelectric material: (**a**) ceramic, (**b**) composite, and (**c**) polymer.

The piezoelectric ceramics used in transducers are shown (Figure 3a). In most previous studies, PZT-4 and PZT-8 have been used extensively [23]. A single lead zirconate titanate (PZT) was not used to design the low-frequency transducer; instead, several PZTs were stacked according to the polling direction as stacking improves the output performance of the transducer [24]. 

In typical examples of transducers operating in the sonar range, a thick piezoelectric material is required to implement the transducer’s resonant frequency at a low frequency. However, due to the manufacturing of piezoelectric materials, it is challenging to implement a thickness of over 2 mm. Therefore, in most studies, low frequencies are realized by stacking piezoelectric materials. When stacking like this, electrodes are stacked in the middle in an interdigital method. Therefore, as the number of electrodes increases, the output efficiency increases. Therefore, a transducer composed of stacked piezoelectric materials has higher output efficiency than a single piezoelectric material transducer.

A piezoelectric composite material that overcomes the disadvantages of piezoelectric materials is shown in Figure 3b. A piezoelectric composite material containing a piezoelectric material and epoxy, which is a polymer, in a specified ratio was designed and implemented. Piezoelectric composite materials have also been applied in laminate techniques for transducers at the sonar range. Piezoelectric composite materials have a wide bandwidth, but are vulnerable to high temperatures, unlike conventional piezoelectric materials [25]. Therefore, piezoelectric ceramic and piezoelectric composite materials are used according to the purpose of the transducer design.

Numerous examples of transducers based on piezoelectric polymers exist [26,27]. Piezoelectric polymers are mechanically flexible compared with piezoelectric materials, as shown in Figure 3c; therefore, they are advantageous when applied to curved structures [12,28]. The transmitting pressure was lower than those of the other transducers. However, compared with piezoelectric materials, piezoelectric polymers exhibit a wide bandwidth performance and are used as receiving sensors.

## 3. Flextensional Transducer

**Figure 4:**
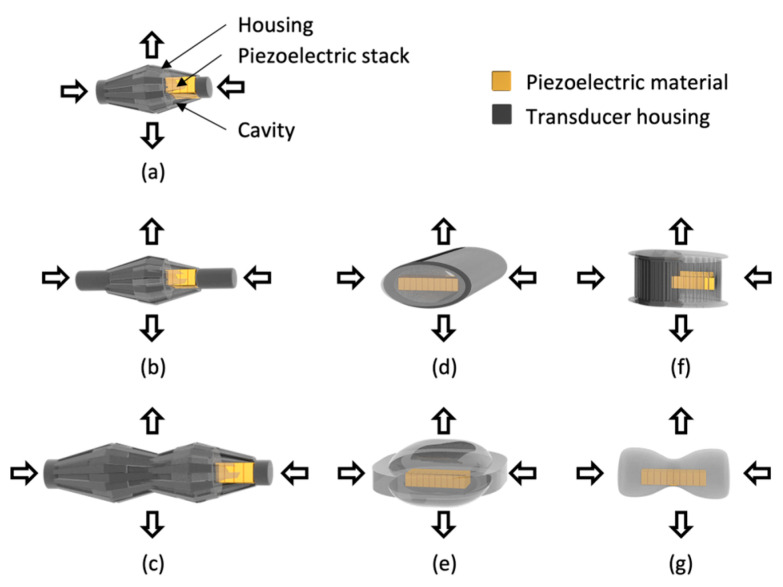
Flextensional transducers: (**a**) class I, (**b**) class II, (**c**) class III, (**d**) class IV, (**e**) class V, (**f**) class VI, and (**g**) class VII.

Flextensional transducers based on piezoelectric ceramics, piezoelectric composites, and piezoelectric polymers have been studied. Generally, a transducer composed of a metal shell, piezoelectric ceramic, and cavity is called a flextensional transducer. Flextensional transducers are classified into seven types according to their shape [29,30]. A schematic of a flextensional transducer is shown in Figure 4. As shown in Figure 4, the flextensional transducer forces the piezoelectric material inside the shell to generate displacements at the left and right ends. Consequently, the displacement is transmitted to the shell, and acoustic signals are generated in the upper and lower directions of the shell.

Flextensional transducers were introduced in the mid-1900s. In the mid-1900s, Brigham introduced class I to class V transducers [31,32]. Class I and class II have similar structures. It has a similar structure, but only the length of the end plate is designed differently. Class I emits sound omnidirectional and is used at a relatively low frequency [33]. Class II emits high sound pressure and is used in a relatively higher frequency band than class I [34]. Class III is a structure that combines two previously introduced class I transducers. It is designed to be used in a broad band by combining transducers with different resonance frequencies [35]. Class IV is a commonly used elliptical transducer, and it is the beginning of a transducer designed for miniaturization while producing high output [36]. Class V is the type known as the moonie and cymbal transducer [37]. Class VI is a transducer similar in shape to class V but designed for use in deeper waters than class V [34]. Class VII emits sound omnidirectionally, making it useful for use at depth [38].

Flextensional transducers, introduced in 1969, were designed for frequencies of 1–2 kHz. Classes I–III transducers have a similar shape, an elliptical shape, and end plates on both sides. Class IV transducers are based on an elliptical shell with a piezoelectric ceramic. Class V transducers are also called cymbal transducers and consist of caps of the same shape at the top and bottom, and a piezoceramic disk is inside the same cap at the top and bottom. In this transducer, the resonance frequency is not adjusted by varying the thickness of the piezoelectric material, but by the design variable of the cap [29].

A transducer is suitable for the target frequency of the barrel flextensional transducer belonging to class I was designed. The performance of the two designed transducers was compared using different materials [39]. The curve is located in the center of the transducer. At this time, the with and without of a change in the resonance frequency of the transducer according to the radius of curvature was identified [40]. Among these transducers, class III, aimed at broadband, combined the longitudinal mode with other modes to spread the radiated sound in all directions [35].

The class IV model of a flextensional transducer with a simple structure consists of a shell and active material. Therefore, it is less affected by an external shell compared with the other models. To improve its performance, a method of adjusting the frequency by inserting a D structure into the coupling part between the active material and shell was introduced [41]. Furthermore, designs other than the shell type have been attempted. A tapered shape for the shell was also designed [42]. In addition, a slot was added to the cap [43]. Most class IV transducer designs focus on increasing the displacement of the end cap. However, attempts have been made to generate a uniform displacement rather than general displacement [44]. Additionally, to overcome the disadvantages of class IV transducers, other modes and resonance modes have been generated. Therefore, when the transmitting voltage response (TVR) (Table 1) is derived, there is no flat section, but more than one mode is created [45].

Among the class IV transducers, a transducer specialized at 900 Hz has also been studied. It consists of a total of six elements, and when composed of one element and an array, all of them were measured in the field. The transducer was designed with two active regions and one inactive region [46].

The active material inside class IV transducers is generally a plate at the center of the shell. However, a shape transformation was made; the material of the active material was not changed. In another study, the active material was arranged in an elliptical shape that is similar to the shell shape [36]. In addition, the active material was composed of PZT, a type of piezoelectric ceramic, and single crystals, and their acoustic characteristics were compared. In this study, a relatively flat section was derived from the frequency response characteristics of low frequency transducers [47].

Class VII transducers, which are an improvement on class IV transducers, are often referred to as dog-bone transducers. To identify the dog-bone transducer’s tendency, the transducer was analyzed using different shell parameters and materials [38]. In the case of existing flextensional transducers, it was a method of fabricating assembling each. In contrast, a new technique has been proposed. Monolithic flexible transducers are more easily accessible than conventional transducers. For this type, increasing the length of the transducer through analysis and measurement can increase the TVR value [48]. In addition, a unique structure was formed for sound transmission. It is composed of a piezoelectric ceramic and spherical cap, and the middle exists as a cavity. The developed transducer exhibited an omnidirectional beam shape but not at a specific frequency. However, if it is omnidirectionally improved, it can be widely used as a next-generation transducer [49].

The aforementioned studies were conducted with a structural focus. They also found that the piezoelectric material present inside the transducer shell is significantly affected by temperature [50]. Moreover, given the change in the piezoelectric ceramic material as well as the temperature effect of the piezoelectric material, the mechanical properties of these devices have been identified [51]. Most of the flextensional transducers are based on piezoelectric ceramics as the internal active material, but other materials have been applied to increase the output pressure of the transducer [52,53]. The maximum output was derived using Terfenol-D, which is based on a permanent magnet-biased driver [52]. The greater the deformation of the transducer shell, the greater the emitted sound; thus, attempts to maximize the movement of the transducer shell are ongoing. Unlike previous studies, a flexible transducer was implemented by applying PVDF (Polyvinylidene fluoride), a piezoelectric polymer, instead of a shell, and the transducer characteristics were evaluated [54].

In addition to research on ceramic materials, research on transducers specialized for long-distance transmission has also been conducted. Transducers specialized for long distances are based on hollow piezoelectric ceramics and have improved output performance owing to the inverse piezoelectric effect [55]. To develop a barrel transducer for stable transmission in the deep sea, an optimization model that applies several variables was proposed [56]. Focusing on long-distance transmission for underwater communication, the Janus transducer consists of two head masses, a piezoelectric ceramic, and an aluminum cylinder. The final model has a diameter of 1.1 m. The developed transducer is helpful in long-distance communication and tomography [40].

According to the TVR characteristics of a previously studied (Table 1) bendable converter, a bendable converter with a narrow bandwidth and relatively wide bandwidth has been proposed [57]. In general, a flexural vibrator has almost no directionality, and thus, it has omnidirectionality. Therefore, it was designed by placing a reflector behind the transducer to obtain an omnidirectional beam pattern and high TVR [58].

There is also a case in which enhancing beam steering rather than the output pressure is the aim. Beam steering based on the flextensional transducer was performed by arranging the shape of the transducer rather than applying a special electrical technique [59]. Six flextensional transducers were placed facing outward to generate a longitudinal mode. An omnidirectional beam pattern was shown by combining the transducers.

Several transducers are combined to achieve beam steering. Subsequently, a transducer based on a single transducer rather than multiple transducers, was designed such that sound waves move in one direction. This transducer is called a class VII transducer and it is a modified form of a class IV transducer [60].

### 3.1. Moonie Transducer

**Figure 5:**
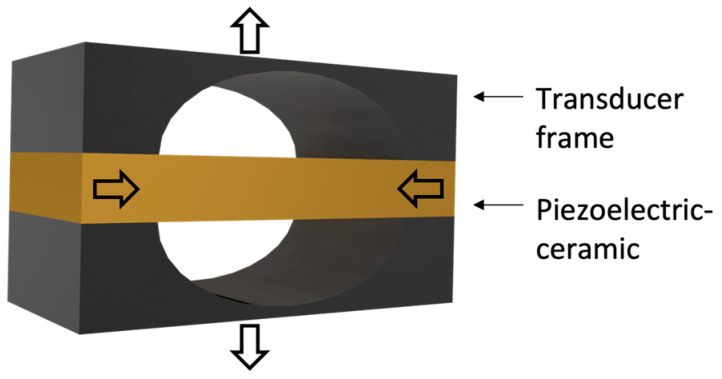
Moonie transducer.

Moonie transducers are flexural transducers that have been studied since the 1990s. The moonie transducer consists of a transducer frame and a piezoelectric ceramic, as shown in Figure 5. When voltage is applied to the piezoelectric ceramic, it moves in the left and right directions. Because the frame of the transducer is affected by the movement of the ceramic, displacement is created in the upper and lower directions of the frame. These deformations allow the moonie transducer to generate sound.

The moonie transducer was also studied in the 1990s. Further analyses of these transducers are required. The moonie transducer has a form similar to that of class V transducers based on metals and piezoelectric ceramics. The metal shell is moved in the z-direction by a piezoelectric ceramic [61]. Therefore, the tendency of the diameter and depth of the cavity between the metal and piezoelectric ceramic has been identified [62].

The performances of similar types of flexible transducers belonging to class V have been studied. The cymbal, grooved moonie, and moonie transducers were compared and analyzed. The effective piezoelectric coefficients of moonie, grooved moonie, and cymbal transducer were derived. As a result, the piezoelectric efficiency was increased by 20% when the end cap of the transducer had a curved shape or a groove [63].

When used underwater, the transducer’s composition material, PZT, must have a high hydrostatic coefficient. Therefore, the hydrostatic coefficient was measured based on the piezoelectric material used in the moonie transducer used as a hydrophone. At last, the moonie transducer has exhibited potential as an underwater acoustic hydrophone because of its relatively high hydrostatic coefficient [64]. The output pressure for the moonie transducer can be seen in Table 2.

### 3.2. Cymbal Transducer

A transducer corresponding to class V flextensional transducers is a cymbal transducer. Cymbal transducers consist of end caps, cavities, and piezoelectric materials. They are structurally simple compared with other transducers and are designed to have high outputs. They are a modified form of the early model moonie transducer, and considerable research on the shape design of the end cap has been conducted. 

**Figure 6:**
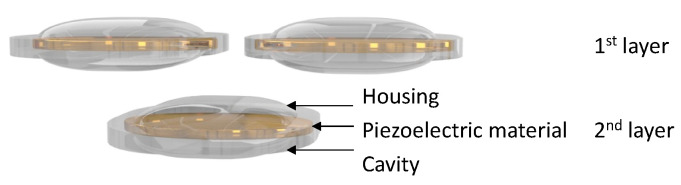
Cymbal transducer array(layer type).

The principle of operation is similar to that of the moonie transducer, and sound is transmitted by moving the end cap up and down while driving the piezoelectric ceramics. Because the resonant frequency is controlled by varying the shape of the end cap, in the case of the cymbal transducer, more focus is placed on the shape of the end cap than on the change in the piezoelectric material. In the case of the cymbal transducer, array research has also been performed as the front and rear surfaces of the transducer are designed to have the same area. When configuring an array from a single transducer, the pitch of the transducer is designed as half wavelength of the resonant frequency. In Figure 6, the resonant frequencies of the first layer and second layer are different to further enhance the output characteristics of the transducer array [14].

**Figure 7:**
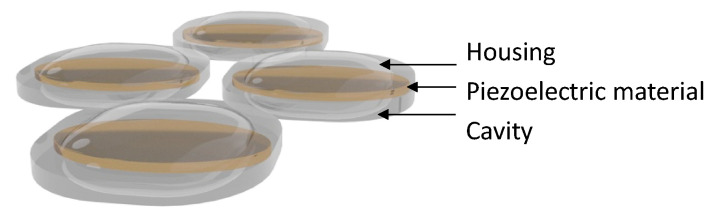
Cymbal transducer array.

To improve the output performance of the cymbal transducer, a multi-frequency array was designed in a planar form, as shown in Figure 7, instead of configuring the transducer layer by layer, as shown in Figure 6. In this method, cymbal transducers with the same resonant frequency are placed on opposite sides. Therefore, transducers having the same resonance have the same pitch by arranging them in half wavelength of the resonant frequency [15]. Research on a single element transducer, transducer array, and arrangements of cymbal transducers using this method is underway.

The cymbal transducer has been used since the 1950s. The presence or absence of a change in the resonant frequency was identified based on the diameter of the cymbal transducer and the type of piezoelectric ceramic inside [65]. The moonie, groove, and cymbal transducers have similar structures, but are designed with different external end caps. External frames play an essential role in generating sound. Therefore, when comparing the movement of the external frame, the thimble transducer showed the best performance [66]. Cymbal transducers can be classified into two types: convex cymbal transducers and concave cymbal transducers. A concave cymbal transducer is designed to withstand high water pressures. However, if the purpose is to transmit over a broad frequency range, a convex transducer is suitable [67]. After the transducer design was determined, research was conducted on the parameters according to the design variables of the cymbal transducer [68,69]. The active material, end cap diameter, end cap material, cavity depth, and resonance frequency of the transducer were determined [70].

The mechanical geometry of flextensional class V transducers did not change much over time until a new model was presented in the 2000s. A donut transducer with a higher electromechanical coupling coefficient than that of a general cymbal transducer was designed [71]. In addition, a change in the transducer shape was observed. Unlike the existing model, the end cap was designed in a spherical shape. In addition to changing the material, a method for changing the resonance frequency by adding a mass to the transducer was proposed [72].

The cymbal transducer has several advantages, and numerous studies on its properties are ongoing. Further, issues that could not be overcome have been identified [73]. To identify the causes of these issues, the electrical characteristics were identified for each frequency of the transducer [74]. Class V transducer arrays present issues regarding yield and ease of manufacture. To overcome these problems, sophisticated simulations are required. Results were derived by considering the cap used when fabricating the transducer, the epoxy used when attaching the active material, and the bonding part [75]. Nonetheless, conjunction issues continue to arise. Sometimes, the transducer does not play its role owing to the adhesive part and can be damaged when a high voltage is applied. Therefore, to solve these issues, a method for designing a ring instead of an epoxy was proposed [76].

The results were compared with the TVR value, a unit used to measure and evaluate underwater transducers. Some data can help designers select materials suitable for cymbal transducers [77]. As research on cymbal transducers became more active, trends in previous studies were also analyzed [78]. Therefore, with increasing interest in nitinol transducers, a new material, nitinol, was used in the design [79]. The temperature dependent mechanical properties of nitinol and general cymbal transducers have been confirmed [80]. In previous studies, the goal was to create two resonant modes without designing a single-transducer broadband system. Different materials of the cymbal transducer were used to create two resonance modes. It was designed as a material with other physical properties [81].

Cymbal transducers, also known as miniaturized transducers, are more suitable for array implementation compared with other low frequency transducers. An example as to why cymbal transducers are better for arrays than other transducers is the tonpilz transducer used in the sonar range. Tonpilz transducers consist of back mass, front mass, and active material. At this time, in the case of the front mass, it is designed in a cone shape to facilitate impedance matching with the medium. Therefore, the front mass radius is designed to be larger than the active material and back mass. On the other hand, the cymbal transducer is configured in the same shape on the top and bottom, and an array can be configured by arranging it with 1/2 wavelength of a desired target frequency. As a result, it is more efficient in terms of spatial arrangement than other transducers

In 1999, cymbal transducer-based array research was introduced. The difference between driving a single transducer and implementing it as an array was shown in [37]. As interest in array implementation has increased, the effect of the cymbal transducer array was verified using finite element analysis [82]. While configuring the transducer as an array, the effect of the metal material of the transducer end cap was also identified [83]. While maintaining the planar array state, a multilayer design of the active material inside the cymbal transducer was attempted [84]. To improve the performance of transducers, research on array configurations has been conducted. Because the optimal element suitable for an array is applied differently according to the purpose of the transducer, after constructing the array, it was improved and formed into a 7 × 7 array [85]. In addition, the 3 × 3 array was comparatively analyzed for transducers using pure materials that were not previously mentioned. The receiving and transmitting characteristics of the first cymbal transducer were derived [86]. An array (1 × 5) hydrophone was introduced to evaluate the performance of a cymbal transducer known as a transducer capable of transmitting and receiving [87]. Between the piezoelectric materials PZT and PNS-PZT, the PNS-PZT material is recommended as a suitable material for hydrophones [88]. In addition, a 1 × 3 receiving array was configured linearly, and the effect of the coating material on the transducer was analyzed [89]. Therefore, the performance of the transducer was determined to be better when implementing an array rather than a single transducer, and an attempt was made to miniaturize the transducer array [90]. In the 2000s, interest in the miniaturization of cymbal transducers and in broadband increased. While implemented as a miniaturization array, it was designed with a relatively small size of 5.5 cm × 5.5 cm [91].

Recently, there has been growing interest in miniaturization and low-profile transducers rather than the output of transducers. Therefore, a smart cymbal panel was developed [92]. To achieve a low-profile transducer, a cymbal array also exists, but a bb-array also exists. The bb array consists of piezoelectric ceramic spheres and is useful for thin profiles [93]. To compensate for the disadvantages of class V transducers, which have a relatively low output pressure compared with class IV transducers, a PCB was inserted into the package [94]. Transducers are not only used underwater but also in air. An air transducer has a shape similar to that of a transducer designed for underwater use and has also been used for energy harvesting [95]. In addition, it has been used in the medical field. The developed transducer consists of a circular or rectangular cymbal transducer to determine the insulin injection effect [96]. Finally, a model with a new shape appeared after the appearance of the moonie and cymbal transducers. The newly introduced model is a wheel type designed to overcome the efficiency degradation caused by the end cap in moonie and cymbal transducers [97]. The output pressure for the cymbal transducer can be seen in Table 3.

## 4. Tonpilz Transducer

**Figure 8:**
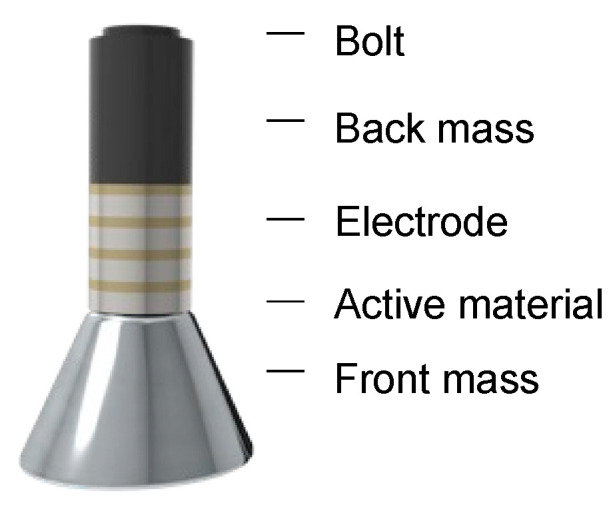
Tonpilz transducer.

Tonpilz transducers, often referred to as mushroom transducers, are configured as shown in Figure 8. They consist of a back mass, active material, and front mass. They are composed primarily of three parts fastened with bolts. The structure of the tonpilz transducer results in improved output characteristics and is more suitable for array implementation rather than for a single transducer. The back mass attached to the back of the device lowers the resonant frequency of the device and focuses the sound forward instead of forward or backward. Piezoelectric ceramics with high piezoelectric coefficients are primarily used as the active materials. To improve their output performance, the devices are stacked.

The front mass is attached to the front part of the device and widens its bandwidth. Numerous studies have been conducted on the shape of the front part of a tonpilz transducer. The transducer was analyzed according to the material and formed of its front part. Initially, the shape of the front part was the same as that of the rear part; however, it was gradually changed to a cone shape to configure a suitable transducer. In studies focusing on widening the transducer bandwidth, the inside of the front part was designed as a hollow shape to reduce the weight of the front part. In addition, a matching layer based on a polymer such as polyurethane was used at the end of the front part to lower the acoustic impedance.

Research has been conducted to improve the performance of tonpilz transducers by changing their active material. Piezoelectric ceramic-based tonpilz designs were the most common type; however, in the 2000s, 2-2, 1-3 piezoelectric composite materials, which are piezoelectric composite materials, were used in tonpilz transducers. The shapes of the front part and the active material were analyzed.

**Figure 9:**
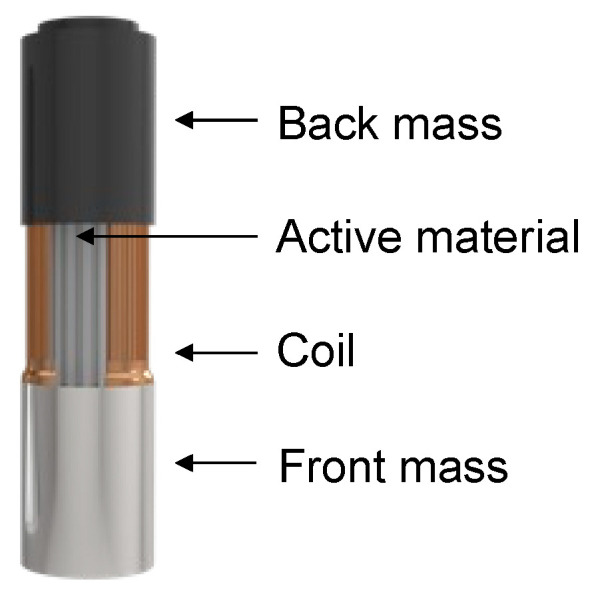
Terfenol-D transducer.

Research has also been conducted to increase the output pressure by changing the active material to a configuration similar to that of a tonpilz transducer. As shown in Figure 9, the reconfigured transducer consists of back mass, active material, and front mass. Terfenol-D was used as the active material, unlike in existing tonpilz transducers. A transducer was constructed using Terfenol-D and a coil; the upper part was designed with Terfenol-D and the lower part was designed with a piezoelectric ceramic. A transducer designed by combining these two methods is called a hybrid tonpilz. Tonpilz transducers appeared, then tonpilz transducers based on Terfenol-D material were developed. Tonpilz transducers increased the output pressure by applying mass to the front and back of the active material, but the bandwidth, which is a vital element of the transducer, could not be overcome. Therefore, researchers studied hybrid tonpilz transducers. The hybrid tonpilz transducer is designed by applying Terfenol-D and piezoelectric materials rather than using Terfenol-D material alone. The hybrid tonpilz transducer has increased bandwidth by about 40% compared to conventional tonpilz transducers. Additionally, hybrid tonpilz transducers can be designed with a lower resonant frequency than piezoelectric materials alone.

Early tonpilz transducers had piston-shaped back masses, an active material, and a head mass. They also produced significant output pressures but with a relatively narrow bandwidth compared with those that were developed more recently [98]. Attempts to transform the head mass shape, one of the main components of the tonpilz transducer, are ongoing. This is because a tonpilz transducer is used underwater; therefore, it must be suitable for such an environment. In a previous study, the end section of the head configuration was designed to be long [99].

Recently, owing to the appearance of a tonpilz transducer designed in a cone shape rather than a piston shape, an analysis was conducted on the flapping mode of the cone shape. The transmission characteristics of the 1st, 2nd, and 3rd modes of the cone shape, and the diameter of the cone shape were compared [100]. A technique for reducing the head mass of a tonpilz transducer has also been studied. To reduce the mass, the shape of the head mass was designed as a void. However, reducing the mass by designing an infinitely large void head mass is not ideal [101]. A hollow head mass did not yield sufficient bandwidth results, and a new head mass design was introduced. In the case of the new head mass, an upper plate was added to the end face of the head mass while maintaining a void state [102]. The new design was used to design a broadband transducer; the head mass of the tonpilz transducer was not changed. Therefore, a center mass was added between the active materials to induce triple resonance in the transducer rather than a single resonance [103]. Sandwich type transducers have also been developed for high-power applications. Materials such as brass and steel are commonly used for the mass of existing transducers, but this transducer was designed based on duraluminum [104].

The tonpilz transducer sets the active material as a variable and compares its output sensitivity [105]. A comparison was made between lead zirconate titanate (PZT), a piezoelectric ceramic used as an active material, and a single crystal [106]. In addition, the shape of the transducer was optimized based on a 2-2 piezoelectric material. Using a 2-2 piezoelectric material, a relatively smaller size than that of other previously designed transducers was derived [107]. Broadband transducers use a 1-3 piezoelectric composite [108].

Electromechanical conversion efficiency is a value according to the conversion of electrical energy and acoustic energy. Therefore, in the case of the Rho group [107], the transducer was designed based on 1-3 piezoelectric composite material. To implement the output pressure of the tonpilz transducer and the broadband transducer, analysis was performed according to the volume fraction of 1-3 piezocomposite. The efficiency was the highest when the PZT-5H volume fraction of piezocomposite was 60%.

To design tonpilz transducers for low frequencies, transducers are stacked with piezoelectric materials. When stacking the transducers, the effect of connecting devices in parallel and series has been demonstrated [109]. Recently, the interest in Terfenol materials has increased. Although there is interest in Terfenol materials, analysis of the device is required before its application to this transducer. Therefore, simulations of the TVR values for Terfenol-D have been performed [110]. Terfenol-D materials have also been introduced as 2nd generation Terfenol composite materials. These materials outperformed single piezoelectric materials and plain Terfenol-D [111]. Furthermore, a high-efficiency transducer can be designed using a magnetic field [112]. The structure of a transducer driven by a magnetic method is described as follows. A copper wire is wrapped around the outside of the transducer, and a cylindrical magnetic bar is inside. Therefore, when power is applied to the wire from the outside, a magnetic field is formed to drive the transducer. 

Recently, a high-power low-frequency tonpilz transducer was constructed using Terfenol-D [113]. In general, tonpilz transducers are realized when Terfenol-D is applied as the active material. Transducers with a high output pressure that can reach a target frequency have also been designed. They were realized using Terfenol-D and a magnetic field [114]. However, Terfenol-D is sensitive to temperature changes. In particular, it is easier to deform than other materials at room temperature. Terfenol-D is expected to be used in the fabrication of devices other than transducers [115].

The sensitivity of tonpilz transducers with a high-pressure output was also tested. Furthermore, both the output and receiving pressures of tonpilz transducers have been measured [116]. However, there are cases in which the same transducer is not used for both transmission and reception, as in previous studies. Transducers usually have both transmit and receive functions. However, due to the structural characteristics of transducers, they can be more specialized for transmitting and receiving. For example, transducers designed to increase transmission characteristics have high transmission characteristics. Take the tonpilz transducer as an example. This transducer consists of a front mass, a back mass, and a piezoelectric material. Since it is applied to the sonar range, it was designed by stacking several layers of piezoelectric materials to use at low frequencies. As several piezoelectric layers are stacked, the resonance frequency decreases, and the output pressure increases. However, piezoelectric materials show a narrow bandwidth in frequency response characteristics as the number of parts increases. If the frequency response characteristic is narrow, the bandwidth that can be transmitted and received is narrowed. In the case of a transducer specialized for reception, it is composed of piezoelectric polymer. The piezoelectric polymer has a wide frequency bandwidth that can be received, but it is challenging to obtain excellent performance in transmission due to its relatively low piezoelectric coefficient. Therefore, to obtain high performance in transmission and reception, it would be adequate to place transducers specialized in transmission and reception, respectively. For example, in underwater environment, a tonpilz transducer is used for transmission and a class V flextensional transducer is used for reception [117].

The tonpilz transducer with an array implementation has also been fabricated. After the fabrication of tonpilz transducers based on a single crystal, those consisting of nine arrays have also been fabricated [118]. An additional analysis for array implementation was also performed, and an efficient head mass design was studied [119].

Similar to flextensional transducers, tonpilz transducers have recently been miniaturized. Tonpilz transducers designed for miniaturization also exhibit flat output characteristics in certain areas of the low frequency band [120]. The output pressure for the tonpilz transducer can be seen in Table 4.

**Figure 10:**
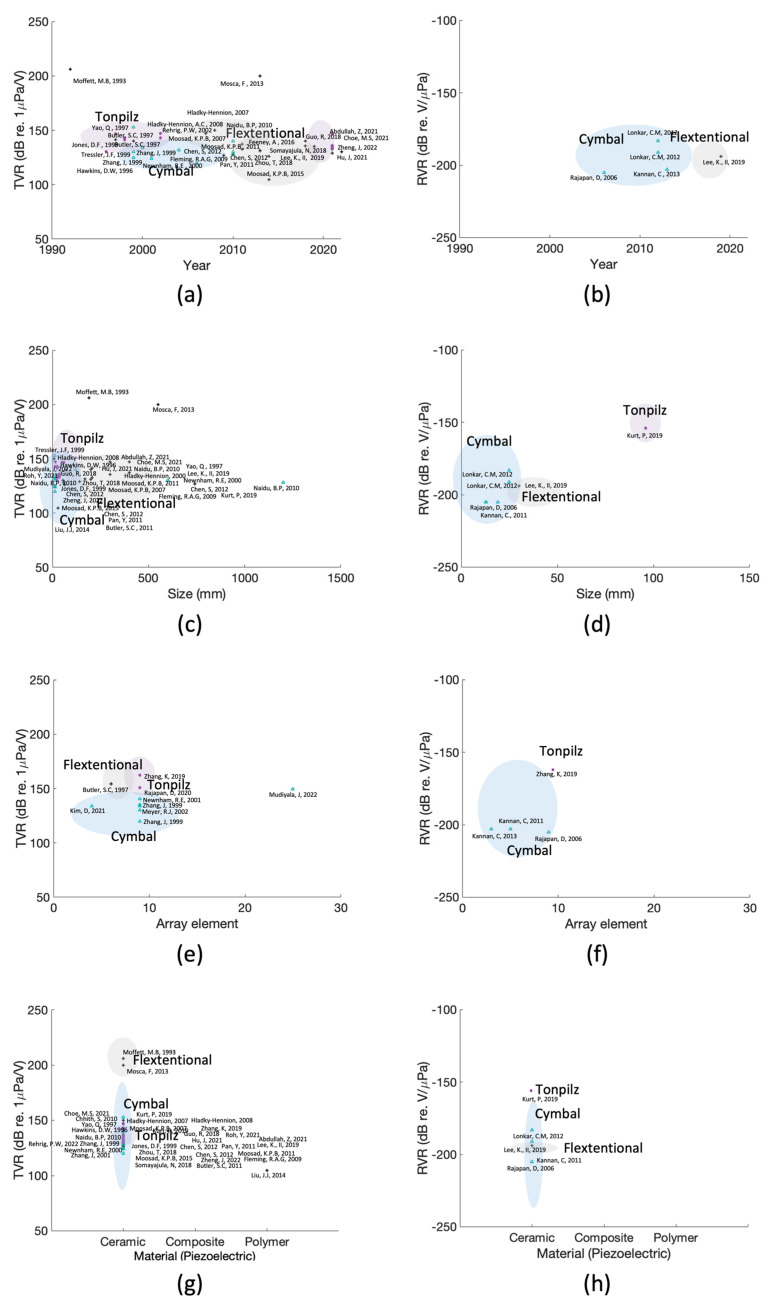
Distribution by transducer type: (**a**) transmitting characteristics by year, (**b**) receiving characteristics by year, (**c**) transmitting response by size, (**d**) receiving response by size, (**e**) transmitting by array, (**f**) receiving by array, (**g**) transmitting response affected by piezoelectric material composition, and (**h**) receiving response affected by piezoelectric material composition [29,30,31,32,33,34,35,36,37,38,39,40,41,42,43,44,45,46,47,48,49,50,51,52,53,54,55,56,57,58,59,60,61,62,63,64,65,66,67,68,69,70,71,72,73,74,75,76,77,78,79,80,81,82,83,84,85,86,87,88,89,90,91,92,93,94,95,96,97,98,99,100,101,102,103,104,105,106,107,108,109,110,111,112,113,114,115,116,117,118,119,120,121].

This can be observed in Figure 10a,b. The results are summarized according to the time when the research was actively conducted and not the year when the transducer first appeared. Each transducer has been studied extensively. An immersion transducer is a device capable of transmitting and receiving. However, referring to previous studies, its use has varied depending on its intended purpose. It has only been used for transmitting, receiving, or both. 

Underwater transducers have recently attracted interest owing to their size. Therefore, visualization based on the size of the transducer is required. Figure 10c,d show the transmission and reception of an existing transducer and the size of the transducer used, respectively. The smallest transducer is the cymbal transducer. However, as other transducers are designed differently for different purposes, their sizes are not limited and vary. If miniaturization is unnecessary, focus is placed on output performance and bandwidth. Finally, miniaturization and high output are challenging to achieve simultaneously. In the future, however, transducers are expected to be designed without trade-offs. Most transducers applied to the sonar range have a resonance frequency under 100 kHz. Below 100 kHz, the length of the wavelength is relatively long. Therefore, the transducer diameter should be increased to emit the output pressure of transducers. However, with the advent of metamaterials, researchers have also focused on miniaturizing sonar range transducers. It is difficult to miniaturize a sonar range transducer while producing an output pressure similar to that of previously developed transducers. To overcome this, the material and design will have to be changed. Then, it will be possible to implement a transducer with a relatively small size and high output pressure.

Several transducers, such as flextensional transducers and tonpilz transducers, are designed based on piezoelectric materials. Therefore, the types of piezoelectric materials used in each transducer were visualized as shown in Figure 10g,h. The shape of the transducer heavily influences flextensional transducers. Therefore, piezoelectric ceramics are widely used as active materials to drive flextensional transducers, and other materials are not widely used. In contrast, tonpilz transducers use other piezoelectric materials to increase the output performance.

Flextensional has been structurally maximizing the transducer movement to improve the transmission characteristics. However, attaching these transducers to underwater vehicles is difficult. Therefore, a transducer with a flat structure, which is the latest transducer type, has become the focus of public attention and research. Therefore, this review, which is based on previous studies, is intended to help the reader understand transducers.

## 5. Conclusions

Most previous studies have focused on low frequency flextensional and tonpilz transducers. However, recently, research on flat and thin low-profile transducers has been conducted. A low-profile transducer can be placed in an array relatively easily and is less constrained by the head mass radius, such as tonpilz transducer. In addition, such transducers offer several advantages, such as reducing resistance to the moving vehicle when used as an active and passive sound absorber by attaching it to an underwater moving vehicle. A low profile is appropriate when these transducers are used for echo control. Underwater sounds can come from several directions. Therefore, studies using sounds at various incident angles are required. Accordingly, the echoes for various incident angles have also been adjusted [121]. Low-profile transducers will be widely used in underwater vehicle detection and concealment technology, and they will continue to be developed to improve their performance.

## 6. Future Direction

Since the 1960s, research on transducers has been conducted. In addition to structural changes in transducers, active materials have been applied to transducers and studied in various ways. Various transducers have been designed and used, in addition to single transducers. Therefore, it is difficult to say that a particular transducer has good performance unconditionally. It would be more accurate to say that a purpose-designed transducer is well-constructed. It should be noted that particular transducers do not exhibit excellent performance unconditionally; however, purpose-designed transducer achieve satisfactory performance in particular situations if they are well constructed.

Therefore, a higher output performance is possible based on the transducers currently under development. However, various transducers will continue to be studied. As research on transducers has progressed in recent years, the external size of transducers has become increasingly small. Until now, thin, and flexible transducers have been challenging to apply because their output pressure is not as good as that in previous studies. However, a relatively thinner, lighter, and more flexible transducer will be developed if this limitation is overcome. 

## Figures and Tables

**Table 1:** Flextensional transducer classifications.

Ref.	Type	Mass	Active Shape	Active Material	Material Type	TVR	RVS	Array	Size
									(mm)
[29]	Flextensional		Plate	Ceramic	PZT			Single	123.9
[30]	Flextensional	Steel		Ceramic	PZT 5H	150 *		Single	400
[33]	Flextensional	Aluminum	Disk	Ceramic	PZT-4	129		Single	141
[35]	Flextensional	Aluminum	Ring	Ceramic	Navy type I	140 *		Single	200
[36]	Flextensional	Aluminum	Arc	Ceramic	PZT-4	135.4		Single	300
[38]	Flextensional	Aluminum	Plate	Ceramic	PZT-8	126.1		Single	64
[39]	Flextensional		Disk	Ceramic	BM400	126.4		Single	
					BM532				
[40]	Flextensional	Aluminum	Disk	Ceramic	Navy type 3	200		Single	550
[41]	Flextensional	Stainless steel	Plate	Ceramic	PZT-8	147 *		Single	300
[42]	Flextensional	Stainless steel	Plate	Ceramic	PZ26			Single	52
[43]	Flextensional	Aluminum	Plate	Ceramic	PZT-4	131		Single	200
									590
[44]	Flextensional	Brass	Plate	Ceramic	PZT-8			Single	44.9
[45]	Flextensional	Aluminum	Plate	Ceramic	PZT-4	131.5		Single	170
[46]	Flextensional	Aluminum	Plate	Ceramic	Navy type 3	141.4		Array	205.7
						154.4			508
[47]	Flextensional	Aluminum	Disk	Ceramic	PMNPT	140 *		Single	72
		Stainless steel			PZT-4				
[48]	Flextensional	Metal	Plate	Ceramic	PZT	150 *		Single	8.75
[49]	Flextensional	Aluminum	Ring	Ceramic	PZT-4	147.2		Single	400
[50]	Flextensional	Aluminum	Plate	Ceramic	PMN-PT			Single	40.1
[51]	Flextensional	Aluminum	Disk	Ceramic	PZT			Single	86
					+Nickel				
					Bismuth				
					Manganese				
[52]	Flextensional	Aluminum	Ring	Magnetostrictive	Terfenol	206		Single	190.5
				Ceramic	PZT-8				
[53]	Flextensional	Aluminum	Plate	Ceramic	Mn:PIN-PMN-PT	130 *		Single	54.6
					PZT-4				
[54]	Flextensional		Plate	Polymer	PVDF	104.7			30
[55]	Flextensional	Steel	Ring	Ceramic	PZT			Single	49
		Duralumin							
[56]	Flextensional	Metallic	Plate	Ceramic	PZT-4			Single	74
[57]	Flextensional	Aluminum	Ring	Ceramic	PZT-4	132.5		Single	206
[58]	Flextensional	Aluminum	Plate	Ceramic	PZT-4	137 *		Single	400
[59]	Flextensional	Aluminum	Ring	Ceramic	Navy type 3	127		Single	740
						154		Array	
[60]	Flextensional	Stainless steel	Plate	Ceramic	PZT-8	127.98		Single	57

*: predicted value (A value derived by extracting data from a graph).

**Table 2:** Moonie transducer classification.

Ref.	Type	Mass	Active Shape	Active Material	Material Type	TVR	RVS	Array	Size
									(mm)
[61]	Moonie	Brass	Disk	Ceramic	PZT-5A			Array	11
		Plastic							
[62]	Moonie	Brass	Disk	Ceramic	PZT-5A			Single	12.7
[63]	Moonie	Brass	Disk	Ceramic	PZT-5H			Single	12.7
					PZT-5A				
					PZT-8D				
[64]	Moonie	Metallic	Ring	Ceramic	PZT			Single	

**Table 3:** Cymbal transducer classification.

Ref.	Type	Mass	Active Shape	Active Material	Material Type	TVR	RVS	Array	Size
									(mm)
[14]	Cymbal	Brass	Disk	Ceramic	PZT-5A	140.7, 149.7		Array	20
[15]	Cymbal	Brass	Disk	Ceramic	PZT-5A	133.7		Array	20
[37]	Cymbal	Titanium	Disk	Ceramic	PZT-5H	125 *		Single	12.7
					PZT-4	135 *		Array	
[65]	Cymbal	Brass	Disk	Ceramic	PZT-4			Single	40
		Stainless steel			PZT-5A				
		Titanium			PZT-8A				
		Aluminum							
		Tungsten			PZT-5H				
[66]	Cymbal	Brass	Disk	Ceramic	PZT-5A			Array	12.7
[67]	Cymbal	Metal	Ring	Ceramic	PKI 552	125		Single	12.7
[68]	Cymbal	Brass	Disk	Ceramic	TSMn-PZT			Single	12.7
[69]	Cymbal	Brass	Disk	Ceramic	PZT-5H			Single	3.2–35
[70]	Cymbal	Tungsten	Disk	Ceramic	PZT-5H			Single	3.2–35
		Molybdenum							
		Brass							
		Steel							
		Titanium							
[71]	Cymbal	Brass	Disk	Ceramic	PZT 5A			Single	25
		Titanium			PZT-4				
		Steel			PZT-8				
		Aluminum			PKI 552				
		Tungsten							
[72]	Cymbal	Kovar	Disk	Ceramic	PZT-5A			Single	12.7
[73]	Cymbal	Kovar	Disk	Ceramic	PZT-5A			Single	12.7
[74]	Cymbal	Kovar	Disk	Ceramic	PZT-5A			Single	12.7
[75]	Cymbal	Titanium	Disk	Ceramic	PZT-5H	124		Array	12.7
					PZT-4				15.9
[76]	Cymbal	Brass	Disk	Ceramic	PZT 402			Single	16.7
[77]	Cymbal	Brass	Disk	Ceramic	PZT-4	140 *		Single	120
						130 *			600
		Al			PZT-5H	128 *			1200
		Stainless steel							6000
[78]	Cymbal	Brass	Disk	Ceramic	PZT	124 *		Single	12.7
						146 *		Array	
[79]	Cymbal	Nitinol	Disk	Ceramic	Sonox P4			Single	12.72
[80]	Cymbal	Silver steel	Disk	Ceramic	Sonox P4			Single	12.71
		Titanium							12.72
		Nitinol							
[81]	Cymbal	Brass	Disk	Ceramic	PZT-5A			Single	12.7
		Steel							
[82]	Cymbal	Metal	Disk	Ceramic	PKI 402	122 *		Array	12.7
[83]	Cymbal	Brass	Disk	Ceramic	PZT-5H	130		Single	12.7
		Titanium			PZT-5A	153		Array	
		Molybdenum			PZT-4				
					PZT-8				
[84]	Cymbal	Titanium	Disk	Ceramic	PKI 552			Array	12.7
[85]	Cymbal	Brass	Disk	Ceramic	Navy type VI	119		Array	12.7
						130			
		Titanium				131			
[86]	Cymbal	Brass	Disk	Ceramic	PZT-4	120	−205	Array	12.7
									12.9
					PZT-5A				19
[87]	Cymbal	Brass	Disk	Ceramic	PZT-5A		−203	Array	13
[88]	Cymbal	Brass	Disk	Ceramic	PZT-5A		−183.2	Single	25
					PNS-PZT		−191.2		
[89]	Cymbal	Brass	Disk	Ceramic	PZT-5A		−203	Array	13
[90]	Cymbal	Brass	Disk	Ceramic	PZT-5H			Single	12.7
								Array	25.4
[91]	Cymbal	Titanium	Disk	Ceramic	PKI 402	134		Array	12.7
[92]	Cymbal	Brass	Disk	Ceramic	PZT-4	130		Array	20
									25
[93]	Cymbal	Titanium	Disk	Ceramic	PKI402	134		Array	12.7
[94]	Cymbal	Steel	Disk	Ceramic	PZT-5H			Single	35
			Plate						
[96]	Cymbal	Titanium	Disk	Ceramic	Navy type I 402	134 *		Array	50.8
			Rectangle						

*: predicted value (A value derived by extracting data from a graph).

**Table 4:** Tonpilz transducer classification.

Ref.	Type	Mass	Active Shape	Active Material	Material Type	TVR	RVS	Array	Size
									(mm)
[99]	Tonpilz	Aluminum	Ring	Ceramic	PZT-4	130		Single	25
		Steel							27
[100]	Tonpilz	Aluminum	Ring	Ceramic	PZT-4	146.8		Single	60
		Brass							
[101]	Tonpilz	Aluminum	Ring	Ceramic	PZT			Single	30
		Steel							
[102]	Tonpilz	Aluminum	Ring	Ceramic	PZT-4	140 *		Single	
		Brass							
[103]	Tonpilz	Aluminum	Ring	Ceramic	PZT-4	136 *		Single	35
		Steel				135 *			
[104]	Transducer	Stainless steel	Ring	Ceramic	PZT-4			Single	25.5
[105]	Tonpilz		Ring	Ceramic	PZT-8	143 *		Single	16
					PMN-PT	147 *			
[106]	Tonpilz		Ring	Ceramic	PMN-PT			Single	
					PZT-8				
[107]	Tonpilz	Aluminum	Ring	Composite	PZT-5H			Single	9–17.4
		Steel			Polymer				
[108]	Tonpilz	Aluminum	Ring	Composite	PZT-5H			Single	13.2
		Steel			Polymer				
[109]	Tonpilz	Aluminum	Ring	Ceramic	NEPEC-6			Single	250
		Steel							
[110]	Tonpilz			Magnetostrictive	Terfenol			Single	
[111]	Tonpilz	Beryllium	Ring	Magnetostrictive	Terfenol			Single	28
[112]	Tonpilz	Aluminum	Ring	Magnetostrictive	Terfenol			Single	168
		Stainless steel							
[113]	Tonpilz	Aluminum	Rod	Magnetostrictive	Terfenol			Single	160
		Stainless steel							
[114]	Tonpilz	Stainless steel	Rod	Magnetostrictive	Terfenol			Single	280
		Magnesium							
[115]	Tonpilz	Metallic	Rod	Magnetostrictive	Terfenol			Single	
[116]	Tonpilz	Aluminum	Ring	Ceramic	PZT-4	136.5	−153.8	Single	96
						138.5	−154.9		
[117]	Tonpilz	Aluminum	Ring	Ceramic	Piezo crystal	151	−163	Array	
		Brass							
[118]	Tonpilz	Beryllium	Ring	Ceramic	Single crystal	146.3 *		Single	
		Tungsten				162.6 *		Array	
[119]	Tonpilz	Aluminum	Ring	Ceramic	PZT-4	146.6		Single	50
		Brass							
[120]	Tonpilz	Aluminum	Ring	Ceramic	PZT-4	132.6		Single	25
						133			
						133.5			
		Stainless steel			PMNPT	133.8			

*: predicted value (A value derived by extracting data from a graph).

## Data Availability

Not applicable.
